# Metabolomic Predictors of Non-alcoholic Steatohepatitis and Advanced Fibrosis in Children

**DOI:** 10.3389/fmicb.2021.713234

**Published:** 2021-08-12

**Authors:** Kattayoun Kordy, Fan Li, David J. Lee, Jason M. Kinchen, Michael H. Jew, Maria Eduarda La Rocque, Sara Zabih, Monica Saavedra, Cora Woodward, Nicole J. Cunningham, Nicole H. Tobin, Grace M. Aldrovandi

**Affiliations:** ^1^Pediatric Gastroenterology, Children’s Hospital of Los Angeles, Los Angeles, CA, United States; ^2^Department of Pediatrics, University of Southern California, Los Angeles, CA, United States; ^3^Department of Pediatrics, University of California, Los Angeles, Los Angeles, CA, United States; ^4^Metabolon, Inc., Morrisville, NC, United States; ^5^Keck School of Medicine, University of Southern California, Los Angeles, CA, United States; ^6^Faculty of Medical Sciences, State University of Rio de Janeiro, Rio de Janeiro, Brazil

**Keywords:** microbiome, pediatrics, NAFLD, NASH, multiomics

## Abstract

Non-alcoholic fatty liver disease (NAFLD) is the leading cause of chronic liver disease in western countries both in children and adults. Metabolic dysregulation associated with gut microbial dysbiosis may influence disease progression from hepatic steatosis to inflammation and subsequent fibrosis. Using a multi-omics approach, we profiled the oral and fecal microbiome and plasma metabolites from 241 predominantly Latino children with non-alcoholic steatohepatitis (NASH), non-alcoholic fatty liver (NAFL), and controls. Children with more severe liver pathology were dysbiotic and had increased gene content associated with lipopolysaccharide biosynthesis and lipid, amino acid and carbohydrate metabolism. These changes were driven by increases in Bacteroides and concomitant decreases of *Akkermansia*, *Anaerococcus*, *Corynebacterium*, and *Finegoldia*. Non-targeted mass spectrometry revealed perturbations in one-carbon metabolism, mitochondrial dysfunction, and increased oxidative stress in children with steatohepatitis and fibrosis. Random forests modeling of plasma metabolites was highly predictive of non-alcoholic steatohepatitis (NASH) (97% accuracy) and hepatic fibrosis, steatosis and lobular inflammation (93.8% accuracy), and can differentiate steatohepatitis from simple steatosis (90.0% accuracy). Multi-omics predictive models for disease and histology findings revealed perturbations in one-carbon metabolism, mitochondrial dysfunction, and increased oxidative stress in children with steatohepatitis and fibrosis. These results highlight the promise of non-invasive biomarkers for the growing epidemic of fatty liver disease.

## Introduction

Non-alcoholic fatty liver disease (NAFLD) is the leading cause of chronic liver disease in western countries both in children and adults (Lazo et al., 2013). The search for reliable non-invasive biomarkers and therapeutic targets has become progressively more urgent as non-alcoholic steatohepatitis (NASH), the most severe form of NAFLD, is quickly becoming the leading cause of liver transplantation (Estes et al., 2018). Intestinal microbiota are implicated as a critical factor in the pathogenesis of NAFLD, but the impact of bacterial metabolism on the liver requires further elucidation. NAFLD is a multi-factorial condition that is intimately linked to obesity and insulin resistance. This metabolic imbalance leads to an excessive accumulation of hepatic fat resulting in non-alcoholic fatty liver (NAFL). In the setting of lipid-laden hepatocytes, fatty acid metabolites cause oxidative stress and a cascade of necroinflammation and fibrosis leading to NASH (Neuschwander-Tetri, 2010). Increasingly, these pathologic processes are being identified in childhood and progress during adulthood. Prevalence varies by ethnicity with Latinos disproportionately affected, possibly due to alterations in hepatic lipid catabolism and genetic polymorphisms (Saab et al., 2016). Non-invasive methods identifying NASH antecedents in childhood provide opportunities to intervene while avoiding confounding factors introduced by aging, alcohol use, concomitant medications, and comorbidities.

Microbiota are implicated in the development of metabolic derangements and inflammation that contribute to hepatic steatosis and progression to steatohepatitis (Ley et al., 2006; Leung et al., 2016; Loomba et al., 2017). Gut microbes dynamically produce, degrade, and modulate metabolites that communicate with the host, influencing the immune response, systemic metabolism, and inflammation (Rooks and Garrett, 2016). Alterations in intestinal microbiota or dysbiosis can lead to changes in insulin sensitivity, free fatty acid production, and disruption of intestinal barrier integrity. The subsequent translocation of bacterial products, including lipopolysaccharide, into the portal circulation and activation of toll-like receptors promotes inflammation and fibrosis of the liver (Henao-Mejia et al., 2012; Pedersen et al., 2016).

Given the importance of dysbiosis and microbial metabolism in NAFLD, we sought to define microbiome-derived biochemicals and metagenomic functions influencing hepatic metabolism and pathologic injury. In this study, we curated the oral and gut microbiota together with serum and fecal metabolites from Latino-predominant children with clinical NAFL and biopsy-proven NASH compared to high and normal weight controls without liver disease. Using a multi-omics approach, we identified microbial-metabolite perturbations influencing a number of pathways including oxidative stress, one-carbon metabolism, tricarboxylic acid (TCA) cycle, lipid and amino acid metabolism. These findings provide key insights for the development of non-invasive biomarkers for the diagnosis of NASH and histologic features, as well as identification of potential therapeutic targets of hepatic injury worthy of further investigation.

## Materials and Methods

### Study Population

Children and adolescents were recruited at Children’s Hospital Los Angeles (CHLA) between September 2016 and June 2017. All participants or their legal guardians provided written informed consent and/or assent. This study protocol was approved by the CHLA Institutional Review Board (approval number: CHLA-15-00395). Four cohorts were recruited: healthy individuals with normal BMI (<85th percentile) and alanine aminotransferase (ALT) < 2x ULN (“normal BMI control”); overweight and obese individuals with BMI ≥ 85th percentile and ALT < 2x ULN (“high BMI control”); overweight and obese individuals with clinically-defined NAFL as elevated ALT ≥ 2x ULN per current practice guidelines (Vos et al., 2017) (ALT ≥ 50 IU/L males, ≥ 44 IU/L females) (“NAFL”); overweight and obese individuals with NASH based on NAFLD activity score (NAS) on clinically-indicated percutaneous liver biopsy as proposed by Kleiner et al. (2005) (“NASH”). Subjects in the latter three groups may have had liver imaging to characterize steatosis. All histology was reviewed and scored by a single pediatric pathologist blinded to clinical and laboratory data. Clinical data was curated from electronic medical records at the nearest time point to specimen collection, including concomitant diagnoses, anthropometrics, medications including any history of vitamin E or metformin, laboratory studies, histology, and imaging. In a few cases, liver transaminases had normalized at the data collection timepoint. There were no dietary restrictions for any subjects, and none of the children received antibiotics for at least 3 months prior to study entry. Children were considered to have type 2 diabetes if they had either HbA1C ≥ 6.5%, fasting serum glucose ≥ 126 mg/dL, or existing clinical diagnosis of type 2 diabetes (Bullard et al., 2013). They were classified as prediabetes if they had either fasting serum glucose between 100 mg/dL and 125 mg/dL or HbA1c ≥ 5.7% and <6.5%.

### Sample Collection

Stool samples, rectal swabs, oral swabs, and peripheral blood were collected. Swabs and stool were frozen neat and stored at −80°C within 6 h of collection. Blood was separated into plasma and PBMC, and was frozen within 6 h of collection.

### Microbial Profiling

Targeted sequencing of the V4 region of the 16S rRNA gene was performed as previously described on all subjects’ fecal and oral samples (Caporaso et al., 2012). DADA2 was used for error correction, sequence inference, and chimera filtering with default settings (Callahan et al., 2016). Taxonomic classification was performed using the RDP naïve Bayesian classifier as implemented in the “dada2” R package. Diversity and ordination analyses were performed using the “phyloseq” R package (version 1.20.0).

### Metabolic Profiling

Non-targeted ultra high-performance liquid chromatography/tandem mass spectrometry (UHPLC-MS/MS) of known biochemicals was conducted on rectal swabs and plasma in subsets of each cohort with well-defined complete clinical and laboratory features [“normal BMI,” *N* = 20; “high BMI,” *N* = 20; “NAFL,” *N* = 25; “NASH,” *N* = 15 (plasma), *N* = 20 (fecal)]. Metabolomics analyses were conducted by Metabolon Inc.,^®^ according to published methods (Ryals et al., 2007; Long et al., 2017). Rectal swabs were normalized to mass.

### Shotgun Metagenomics

To further characterize functional pathways potentially related to metabolites and microbiota, whole genome shotgun sequencing was performed from fecal samples in a subset of NASH (*N* = 20) and control (*N* = 20) subjects who had metabolomic profiling to an average depth of 38,984,068 ± 12,462,099 reads per sample. Adapter trimming and quality filtering were performed using trim galore, host sequences were removed using kneadData, and taxonomic classification was performed with Kraken (v0.15-beta). Functional profiling was performed using HUMAnN2. Differentially abundant pathways were identified using the “DESeq2” R package (version 1.16.1). FishTaco (version 1.1.1) was used to identify taxonomic drivers of functional shifts in the NASH microbiome.

### Statistical Analysis

Zero-inflated negative binomial (ZINB) regression (“pscl” package version 1.4.9) and ordinary linear regression were utilized to identify specific taxa and compounds associated with variables of interest. All *p*-values were corrected for multiple testing using the Benjamini–Hochberg FDR method. An adjusted *p*-value < 0.1 was considered significant. Random forests classification models were built using the “randomForest” R package (version 4.6-12) with genus-level relative abundances or normalized metabolite levels, age, sex, and BMI as covariates. Random forests is an ensemble classifier method that is relatively robust to outliers and overfitting, and can be used to perform feature selection on the basis of importance scores (Statnikov et al., 2013). Taxa present at less than 1% relative abundance in less than 10% of the samples were excluded to reduce the size of the feature set. Mean importance scores were computed for each covariate using 100 permutations and 10,000 trees per forest. A sparse model was constructed by including all covariates with a mean importance greater than 0.001, up to a maximum of 30. Receiver-operator characteristic (ROC) curves were drawn using the “ROCR” R package (v1.0-7). RF model performance was measured *via* several methods including classification accuracy, area under the ROC curve (AUC) for binary classification models, and Matthews correlation coefficient (MCC) for both binary and multiclass models. Binary and multiclass Matthews correlation coefficients (MCC) were computed using the “mltools” R package (Gorman, 2018).

### Multi-Omics Modeling

Multi-omics models for disease cohort and histology findings were built by combining features from the 16S rRNA profiling of the gut and oral compartments as well as metabolic profiling from rectal swabs and plasma on a subset of *n* = 79 subjects (Table 1) for whom all four assays were performed. Genus-level relative abundances and normalized metabolite levels were used as in the separate models, and age, sex, and BMI were included as covariates.

**TABLE 1:** Baseline characteristics and multi-omic sampling for the 241 subjects enrolled in the study.

	Normal BMI	High BMI	NAFL	NASH	*p*-value
**Demographics**					
Total N	54	80	86	21	
Age, median (range)	12 (5–20)	11 (6–18)	12 (7–19)	13 (8–21)	0.008
5–11 (%)	24 (44.4)	47 (58.8)	36 (41.9)	7 (33.3)	
12–17 (%)	28 (51.9)	32 (40.0)	36 (53.5)	12 (57.1)	
18–21 (%)	2 (3.7)	1 (1.2)	4 (4.7)	2 (9.5)	
Male N (%)	24 (44.4)	45 (56.2)	53 (61.6)	14 (66.7)	0.18
Hispanic N (%)	47 (87.0)	68 (85.0)	78 (90.7)	21 (100)	0.23
**Anthropometric, median**					
BMI (kg/m^2^) (range)	18.2 (13.2–24.0)	28.4 (19.2–55.1)	32 (18.8–48.4)	33 (21.5–51.7)	<0.001
BMI z-score (range)	0.17 (−1.78–1.27)	2.99 (0.60–16.90)	3.15 (0.58–6.05)	3.12 (1.76–5.73)	<0.001
**Liver enzymes, median (range)**					
ALT^b^ U/L	26 (18–41)	29 (8–48)	65 (8–391)	122 (19–982)	<0.001
AST^c^ U/L	32 (21–52)	30.5 (14–49)	41.5 (15–174)	64 (23–639)	<0.001
AST/ALT	1.29 (0.59–2)	1 (0.57–2.38)	0.66 (0.3–1.88)	0.67 (0.4–1.26)	<0.001
**Laboratory studies**					
Hemoglobin A1C, mean	5.55	5.5	5.5	5.7	0.47
Triglycerides mg/dL, median	67.5	133	142.5	141	<0.001
HDL^d^ mg/dL, median	47	38	40	38	0.006
Hypertriglyceridemia and abnormal HDL, N (%)	1 (1.8)	29 (36.3)	33 (38.4)	11 (52.4)	<0.001
**Clinical N (%)**					
Prediabetes	2 (3.7)	17 (21.3)	22 (25.6)	4 (19)	0.004
Type II DM	0 (0)	2 (2.5)	0 (0)	5 (23.8)	<0.001
Vitamin E	0 (0)	1 (1.3)	15 (17.4)	7 (33.3)	<0.001
Metformin	0 (0)	3 (3.8)	1 (1.2)	6 (28.6)	<0.001
**Multi-omic Sampling (total N)**					
16S^e^, Oral Swab	54	80	86	21	241
16S, Rectal Swab	54	80	86	21	241
Shotgun Metagenomics, Rectal Swab	20	0	0	20	40
Metabolomics, Plasma	20	20	25	15	80
Metabolomics, Fecal	20	20	25	20	85
Multi-omics, combined	19	20	25	15	79

*^*a*^SD, standard deviation; ^*b*^ALT, alanine aminotransferase; ^*c*^AST, aspartate aminotransferase;*

*^*d*^HDL, high-density lipoprotein;^*e*^16S, 16S rRNA gene sequencing.*

## Results

### Progressive Metabolic Derangements in Children With Worsening Non-alcoholic Fatty Liver Disease

Two-hundred forty-one predominantly Latino subjects (89%), 56% male, ages 5–21 years were enrolled in the study. The normal body mass index (BMI) (*N* = 54), high BMI (*N* = 80), and NAFL (*N* = 86) groups had median ages of 12, 11, and 12 years, respectively. The NASH (*N* = 21) group was slightly older with a median age of 13 years (*p* = 0.008). While controlling for BMI, the degree of increased transaminases and prevalence of diabetes significantly increased in subjects with worsening liver disease (*p* < 0.001). A striking 23% of children with steatohepatitis had diabetes mellitus (hemoglobin A1C ≥ 6.5) and an additional 23% had prediabetes, which corroborate reports of children with type 2 diabetes having an increased likelihood of NASH (Newton et al., 2016). All subjects with NASH were Latinos with a median ALT of 122 U/L (range 19-982 U/L), significantly higher than the NAFL group (median ALT 65 U/L) and control groups (median ALT 26-29 U/L). The AST/ALT ratio decreased to 0.66–0.67 with fatty liver disease compared to controls without liver injury (1.0–1.29) (*p* < 0.001).

Biopsy-proven NASH subjects had a median NAFLD Activity Score of 5 (range 3–7). They all had portal inflammation and fibrosis: two-thirds with mild fibrosis (F 1–2), one-third with moderate to advanced fibrosis (F 3–4), and one 8-year old subject had cirrhosis. Two-thirds of high BMI and NAFL subjects and 78% of NASH subjects had dyslipidemia, either isolated hypertriglyceridemia or low HDL or both. A few subjects with NAFL were empirically treated with vitamin E (17.4%) and/or metformin (1.2%), while a larger proportion of children with NASH received these medications (33.3% and 28.6%, respectively) at some point during their treatment history. Table 1 shows the demographic, clinical and biochemical characteristics of the four cohorts. Table 2 provides detailed histology on the 23 subjects with liver biopsies, 21 of whom were diagnosed with NASH and 2 with NAFL.

**TABLE 2:** Histology of a subset (*N* = 23) of subjects with NAFLD on liver biopsy.

Histology	Binned variables	*N*
**NAS^a^**		
0–2	Mild	2
3–4	Advanced	7
≥5		14
Median (range)		5 (2–7)
**Steatosis**		
0	1	1
1		7
2	2	10
3	3	5
Median (range)		2 (0–3)
**Lobular Inflammation**		
0	Mild	1
1		8
2	Advanced	14
3		0
Median (range)		2 (0–2)
**Portal Inflammation**		
Yes	Yes	22
No	No	1
**Ballooning**		
0	Mild	0
1		15
2	Advanced	8
Median (range)		1 (1–2)
**Fibrosis**		
0–2	Mild	15
3–4	Advanced	8
Median (range)		2 (1–4)
**Cirrhosis**		
Yes	Yes	1
No	No	22

*^*a*^NAS, NAFLD Activity Score.*

### Oral and Fecal Microbial Signatures of Progressive Liver Disease

**FIGURE 1:**
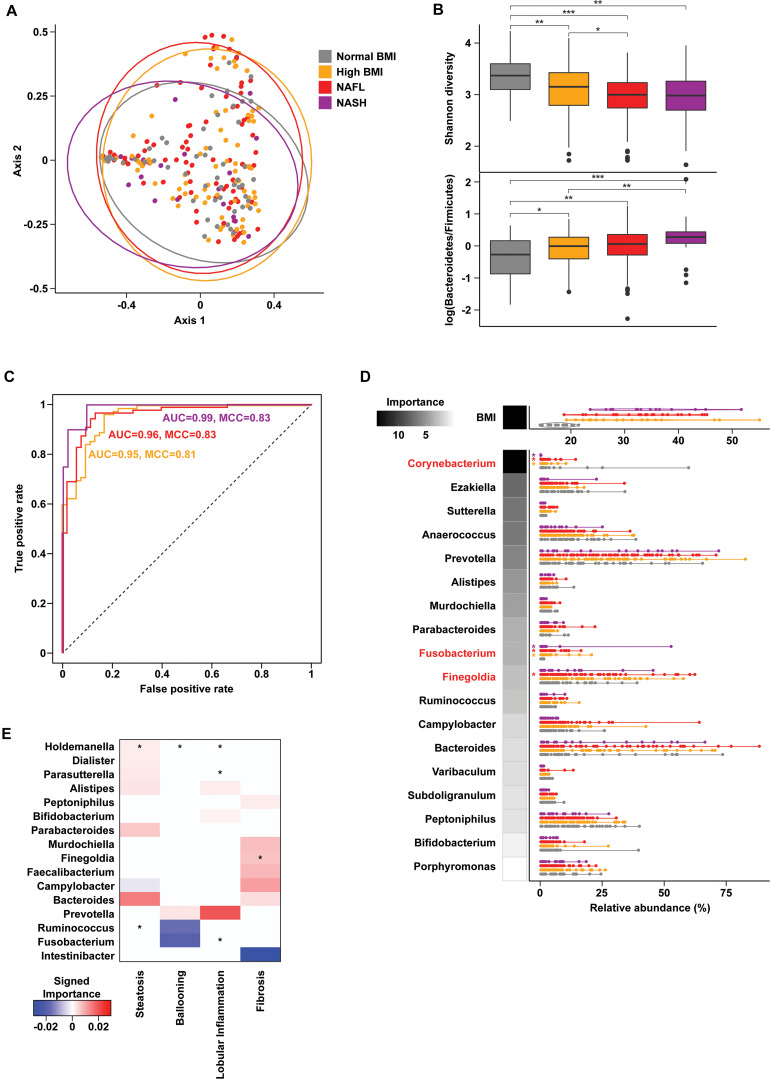
Gut microbiome composition in progressive fatty liver disease. **(A)** Principal coordinates analysis (PCoA) plot of gut microbiota composition using Bray-Curtis distances. Ellipses show 95% confidence regions. **(B)** Boxplots show significantly decreased Shannon diversity (KW *p* = 3.06e-06) (top) and increased log-transformed *Bacteroidetes* to *Firmicutes* ratio (KW *p* = 0.001) (bottom) with advancing liver disease. Pairwise comparisons using a Wilcoxon test are indicated along the top. *p < 0.05, **p < 0.01, and ***p < 0.001. **(C)** Receiver operating characteristic (ROC) curves of binary random forests (RF) classification models for each group as indicated vs. “normal BMI” baseline. **(D)** Distribution of BMI and genus-level relative abundances for the features selected in the multiclass RF model (violin plots). RF model importance values are shown as shaded boxes. Red text denotes genera identified as significantly altered in zero inflated negative binomial (ZINB) regression models. **(E)** Importance values for RF models of liver histology findings. Positive (red) and negative (blue) values indicate genera that are increased and decreased with higher grades of each finding, respectively. Asterisks (*) denote genera that are also significant (*p* < 0.1) in ZINB regression analyses.

Gut microbiome profiling using 16S rRNA sequencing revealed weak but statistically significant segregation by cohort (PERMANOVA R2 = 0.02, *p* = 0.009, Figure 1A and Supplementary Table 1). Advanced liver disease was associated with an increasing *Bacteroidetes:Firmicutes* ratio (KW *p* = 0.001) and decreasing alpha diversity (KW *p* = 3.06e-06) (Figure 1B). Random forests classification with genus-level relative abundances as well as age, sex, and BMI as covariates revealed a marginal ability to separate the four cohorts [46.7% accuracy, Matthew’s correlation coefficient (MCC) = 0.27], but pairwise classification models against the normal BMI baseline achieved excellent accuracies (Figure 1C). At the genus level, the relative abundance of *Corynebacterium* (*Z* = −6.75, padj = 1e-09) was significantly decreased in NASH subjects relative to normal controls, while relative abundances of *Fusobacterium* (*Z* = 4.74, padj = 7.24e-05) and *Finegoldia* (*Z* = 2.79, padj = 0.059) were increased in NASH (Figure 1D). *Corynebacterium* relative abundance was also negatively associated with levels of ALT, AST, and GGT (Supplementary Table 2). A similar analysis at the species level showed slightly better multiclass predictive accuracy (47.5%, MCC = 0.27, Supplementary Figure 1), but failed to identify any specific bacterial species after correction for multiple comparisons.

Oral microbiome profiling also showed distinct communities by cohort (PERMANOVA R2 = 0.02, *p* = 0.003, Supplementary Figure 2 and Supplementary Table 1), but did not reveal any significant differences in the *Bacteroidetes:Firmicutes* ratio or alpha diversity. Multiclass classification using oral microbial composition as well as age, sex, and BMI performed better than gut microbial composition at cohort separation (54.1% accuracy, MCC = 0.36), but no genera were significantly different in NASH. Intriguingly, species-level analysis of the oral microbiome showed higher predictive accuracy (60.3%, MCC = 0.44, Supplementary Figure 2) and identified a number of significantly increased bacterial species including unclassified *Streptococcus* (*p* < 0.01) and *Haemophilus influenzae* (*p* = 0.09) in NASH.

Histologic steatosis was associated with increases of fecal *Holdemania* (*Z* = 14.93, padj < 2.2e-16) and oral *Alloprevotella* (*Z* = 4.27, padj = 0.001), *Prevotella* (*Z* = 2.69, padj = 0.07), and *Veillonella* (*Z* = 3.62, padj = 0.007). Subjects with advanced fibrosis had increases in fecal *Finegoldia* (*Z* = 2.67, padj = 0.072) and *Faecalibacterium* (*Z* = 2.76, padj = 0.069) (Figure 1E), and oral *Veillonella* (*Z* = 3.62, padj = 0.007) and *Prevotella* (*Z* = 2.69, padj = 0.07) (Supplementary Figure 2).

### Microbial Metagenome-Encoded Metabolic Alterations in NASH

Recent work has highlighted the importance of understanding both the functional shifts as well as taxonomic alterations in various disease processes. We performed shotgun metagenomic sequencing on a subset of normal BMI controls (*N* = 20) and NASH (*N* = 20) subjects to characterize the taxonomic and functional differences in intestinal microbial communities. Only a few differences in microbial composition were observed (Supplementary Figure 3 and Supplementary Table 4; PERMANOVA R2 = 0.05, *p* = 0.09), however, changes in specific bacteria (Supplementary Table 4) were associated with significant enrichment of genes associated with lipid metabolism, amino acid metabolism, carbohydrate metabolism, and lipopolysaccharide (LPS) biosynthesis. These functional shifts were associated with predominant increases of *Bacteroides* species as well as decreases in *Akkermansia muciniphila*, *Anaerococcus prevotii*, *Corynebacterium sp.*, and *Finegoldia magna* (Supplementary Figure 4).

Differential abundance analysis of functional metagenomic pathways using a negative binomial model suggested downregulation of L-methionine (padj = 0.18), L-threonine (padj = 0.18), sulfur amino acid biosynthesis pathways and anaerobic metabolism (Supplementary Table 5).

### Metabolic and Multi-Omic Signatures of Progressive Fatty Liver Disease

**FIGURE 2:**
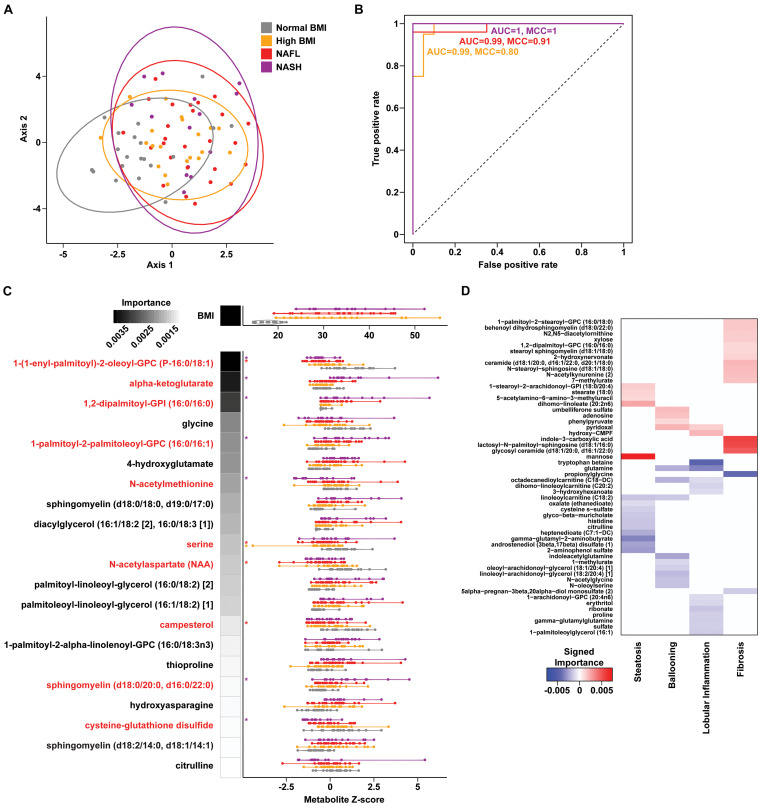
Plasma metabolite signatures of progressive fatty liver disease. **(A)** T-distributed stochastic neighbor embedding (t-SNE) plot of plasma metabolite profiles. Ellipses show 95% confidence regions for each cohort. **(B)** Receiver operating characteristic (ROC) curves of binary RF classification models for each group as indicated vs. “Normal BMI” baseline. **(C)** Distribution of BMI and metabolite values for the features selected in the multiclass RF model (violin plots). RF model importance values are shown as shaded boxes with darker colors denoting higher mean importance. Red text denotes metabolites identified as significantly altered in linear regression models. **(D)** Heatmap of importance values for RF models of liver histology findings. Positive (red) and negative (blue) values indicate metabolites that are increased and decreased with higher grades of each finding, respectively.

**FIGURE 3:**
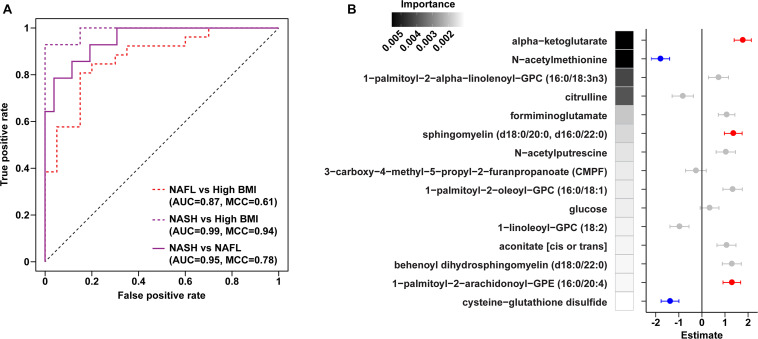
Plasma metabolite binary classification models against “high BMI” or “NAFL” baselines. **(A)** ROC curves of binary RF models for each comparison as indicated. **(B)** Forest plot of linear regression coefficients (“Estimate”) for features selected in the binary NAFL vs. NASH plasma metabolite RF model. Positive (red) and negative (blue) coloring denotes metabolites significantly increased and decreased in NASH relative to NAFL, respectively. Error bars show standard errors of the regression coefficients. RF model importance values are shown as shaded boxes.

To assess changes associated with NAFL and NASH, metabolomics analysis of plasma and rectal swabs was performed on a subset of subjects (*N* = 85) drawn from all four cohorts (20–25 subjects per cohort). We identified and quantified 832 and 730 compounds in plasma and fecal samples, respectively. t-SNE projection and PERMANOVA of plasma metabolite profiles revealed moderate separation between the four cohorts (Figure 2A and Supplementary Table 6, PERMANOVA R2 = 0.054, *p* = 0.001) as well as a significant BMI-mediated effect (PERMANOVA R2 = 0.027, *p* = 0.001). Random forests classification using normalized plasma metabolite levels as well as age, sex, and BMI as covariates yielded a predictive accuracy of 61.3% (MCC = 0.48) whereas random chance would be expected to yield 25% (Figures 2B,C). To isolate the effect of liver disease from BMI, we also constructed binary classifiers for NAFL (80.4% accuracy, MCC = 0.61) and NASH (97% accuracy, MCC = 0.94) compared to the high BMI control and NASH vs. NAFL (90% accuracy, MCC = 0.78) (Figure 3). The top biochemicals contributing to group separation were predominantly lipids and amino acids.

**FIGURE 4:**
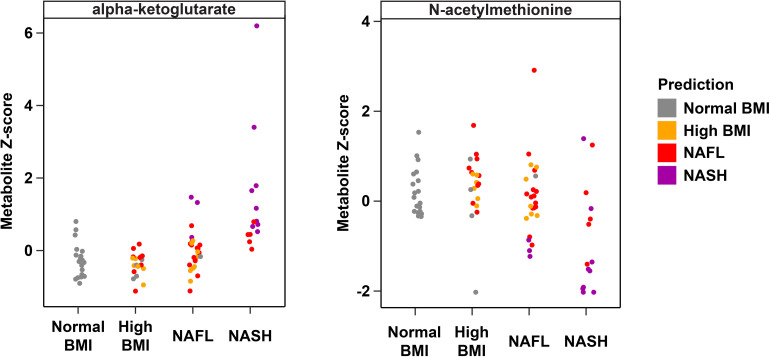
Plasma metabolite drivers of disease cohort misclassification. Ground truth classes based on original clinical diagnosis are shown along the x-axis, predicted classes are shown as the indicated colors, and each point represents one individual. For example, three subjects in the NAFL group who were misclassified as NASH (purple points in the “NAFL” x-axis group) show elevated levels of alpha-ketoglutarate consistent with the NASH profile.

Metabolites significantly associated with steatohepatitis included biochemicals involved in metabolism for energy, importantly alpha-ketoglutarate, glutathione synthesis, markers of oxidative stress, methionine, phosphatidylinositol, phosphatidylcholine, amino acids, tryptophan, sphingolipids, and purine (Supplementary Figure 5). Metabolites associated with NAFL included those involved with metabolism of primary bile acid, fatty acid, glutathione, and tryptophan. Interestingly, two subjects with a clinical diagnosis of NAFL were misclassified by the model as NASH driven by increased abundance of alpha-ketoglutarate and N-acetylmethionine (Figure 4).

Downregulation of biomarkers of one-carbon metabolism in the mitochondria was observed in both our metagenomic and metabolomics analysis of NASH subjects. Amino acids that feed one-carbon metabolism serine and glycine were significantly decreased in the NASH cohort (*p* = 0.002) and methionine-derivatives choline and cysteine were also decreased (Figure 2C). Oxidative stress is reflected by significant alterations in γ-glutamyl-amino acids, cysteine-glutathione disulfide, thioproline, serine, glycine and N-acetylmethionine in our NASH cohort. Furthermore, four plasma metabolites were predictive across the multiclass, NASH vs. high BMI control, and NASH vs. NAFL models: alpha-ketoglutarate, cysteine-glutathione disulfide, N-acetylmethionine, and citrulline. These metabolites were not present in the model separating NAFL from high BMI control (Supplementary Table 7), and were also significantly associated with levels of AST, ALT, and GGT (Supplementary Table 8).

**FIGURE 5:**
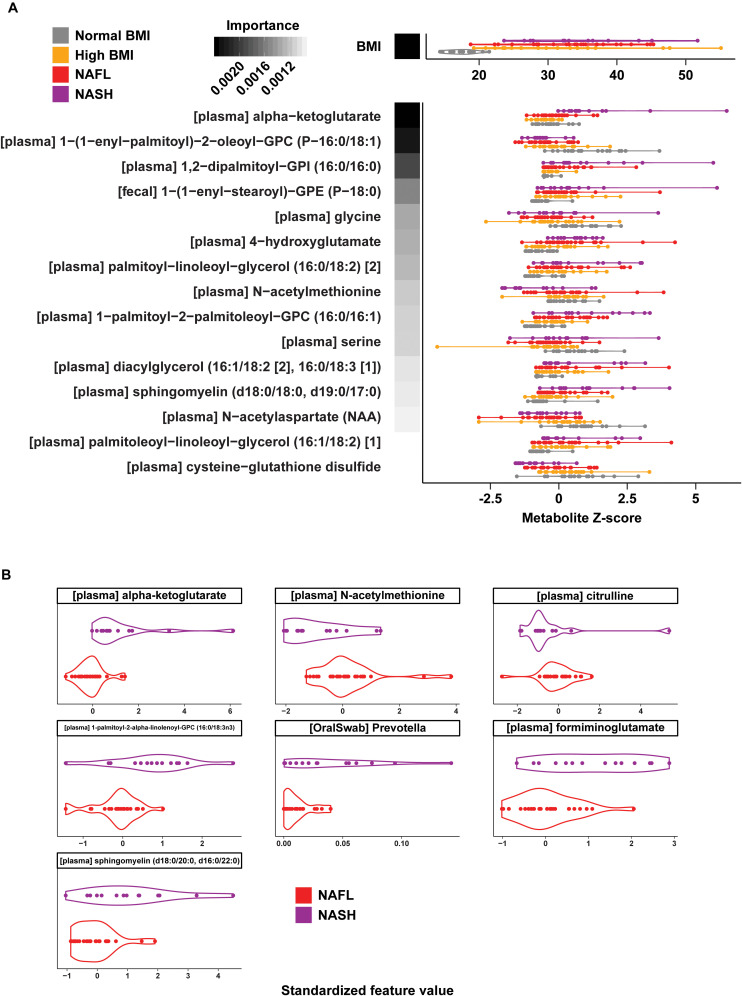
Multi-omic classification models using metabolomics and 16S microbiome profiling data. **(A)** Distribution of BMI and standardized values for the features selected in the multiclass multi-omic RF model (violin plots). RF model importance values are shown as shaded boxes with darker colors denoting higher mean importance. Text in brackets denote the source of each selected feature (plasma or fecal metabolite). **(B)** Distribution of standardized values for the features selected in the NAFL vs. NASH multi-omic RF model (violin plots). Text in brackets denote the source of each selected feature.

Similar analyses using fecal metabolite profiles showed generally weaker classification performance for the multiclass (50.6% accuracy, MCC = 0.33), NAFL (69.6% accuracy, MCC = 0.37), and NASH (74.4% accuracy, MCC = 0.49) models (Supplementary Figures 6, 7). A multi-omic model including gut and oral microbial profiles as well as plasma and fecal metabolites yielded slightly improved predictive accuracy for disease cohort (overall accuracy of 64.6% vs. 61.3% for plasma metabolites alone) (Figure 5). This improvement was likely due to the inclusion of a single fecal metabolite 1−(1−enyl−stearoyl)−GPE (P−18:0), an antioxidant with a role in lipid metabolism (Ilievski et al., 2016; Rebholz et al., 2018; Gu et al., 2020), which increased with disease progression. Analogous binary classifiers for NAFL and NASH vs. high BMI controls, and for NASH vs. NAFL also yielded small improvements in model performance. Interestingly, the relative abundance of *Prevotella* in the oral compartment was an important predictor for NASH, consistent with previous reports of elevated oral *Prevotella* in adults with NASH (Bashiardes et al., 2016; Figure 5).

### Metabolites Predict Steatosis, Lobular Inflammation, and Fibrosis in Fatty Liver Disease

A set of random forest (RF) classification models for steatosis, lobular inflammation, and fibrosis using the plasma metabolite profiles as predictors of the histologic findings were constructed (Supplementary Table 9). For steatosis, 15 metabolites were selected *via* cross-validation including mannose sugar monomer, peptide (gamma-glutamyl amino acid), androgenic steroids (androstenediol), and a number of components in lipid metabolism (Figure 2D). Fourteen important predictors for lobular inflammation included amino acids (tryptophan, glutamine), vitamin B6 metabolism, and components of fatty acid and lysophospholipid metabolism (Figure 2D). These RF models achieved a high predictive accuracy of 93.7% with MCC = 0.909 and 0.870 for steatosis and lobular inflammation, respectively. Only a single mild steatosis subject, driven by lipids heptenedioate (C7:1-DC) and linoleoylcarnitine (C18:2), and a single mild lobular inflammation subject, driven by tryptophan betaine and sulfate, were misclassified with higher grades of these histological features (Supplementary Figure 8).

Fibrosis had a metabolic signature consisting of 16 metabolites with high predictive accuracy (93.7% accuracy, MCC = 0.882) with biochemicals relevant to inflammation (Figure 2D). Overall, subjects with advanced fibrosis had significantly higher plasma lipids—in particular, sphingolipids and ceramides—and indole-3-carboxylic acid compared to individuals with mild fibrosis (0–2). Furthermore, sphingolipids were important predictors for both fibrosis and NASH: behenoyl dihydrosphingomyelin was present both in the RF model of fibrosis and the model differentiating NASH and NAFL; N-stearoyl-sphingosine and glycosyl N-stearoyl-sphingosine was predictive of fibrosis and separated NASH from high BMI controls. Only a single subject labeled with mild fibrosis (0–2) was misclassified as advanced fibrosis, driven by increased ceramide (d18:1/20:0, d16:1/22:0, d20:1/18:0) (Supplementary Figure 8).

## Discussion

Fatty liver disease, the hepatic manifestation of metabolic syndrome, may begin in childhood and can progress to cirrhosis and end-stage liver disease in adulthood, posing a serious public health threat. The ability to distinguish these individuals and intervene prior to the development of advanced liver disease is critically important. Our analyses of oral and rectal microbial communities, metagenomics and metabolomics in well-characterized Latino-predominant children comprehensively identify predictors of NASH and histologic injury. We identified potential microbial contributions to altered functional pathways and characterized perturbations of metabolites likely contributing to disease pathogenesis through oxidative stress. Furthermore, we identified panels of plasma metabolites that can non-invasively identify NASH and fibrosis with high predictive accuracy similar to the accuracy of complex multi-omic models and performing better than taxonomy alone. These findings increase our understanding of the pathogenesis of pediatric fatty liver disease and suggest potential non-invasive diagnostic markers and therapeutic targets for NASH and fibrosis.

The progressive dysbiosis and lower alpha diversity in our cohort of children with NASH correlated with increasing dyslipidemia, insulin resistance, fatty liver disease severity and advanced fibrosis. NASH was associated with significant increases in oral *Streptococcus* and gut *Fusobacterium* with concomitant decreases in gut *Corynebacterium.* Interestingly, *Corynebacterium* produces lipases that release antibacterial free fatty acids from host lipids which ultimately regulate surrounding bacterial species (Bomar et al., 2016); its depletion in NASH may contribute to distinct alterations of the surrounding microbial community. Oral dysbiosis is emerging as a potential source of systemic inflammation and cirrhosis (Qin et al., 2014; Acharya et al., 2017). Indeed, advanced fibrosis in children was associated with increased oral *Veillonella* and *Prevotella*. In adults with cirrhosis, oral *Veillonella* and *Prevotella* are presumed to seed the intestinal microbiome and are correlated with progression of fibrosis (Qin et al., 2014).

In children with NASH, we observed increases in genes associated with upregulation of lipid, carbohydrate and amino acid metabolism and LPS biosynthesis. Taxon level contributions to these shifts were driven by increases of *Bacteroides* and depletion of *Akkermansia, Anaerococcus, Corynebacterium* and *Finegoldia*. The upregulation of LPS in children with NASH has been reported by others (Alisi et al., 2010; Schwimmer et al., 2019), and linked with progression to NASH *via* stimulation of toll-like receptor (Ruiz et al., 2007; Verdam et al., 2011; Roh and Seki, 2013; Sharifnia et al., 2015; Carpino et al., 2019). We hypothesize that early microbial predominance of *Bacteroides* in childhood along with loss of *Corynebacterium, Akkermansia* and *Anaerococcus* with their role in stabilizing the surrounding bacterial communities and the gut epithelial barrier may lead to functional changes that enable the progression of fatty liver disease.

Untargeted metabolomics profiling, in conjunction with metagenomic analysis, has shown significant promise as biomarkers of fatty liver disease progression in other pediatric cohorts (Zhu et al., 2013; Del Chierico et al., 2017; Khusial et al., 2019). We identified plasma metabolite signatures that distinguished those who had progressed to NASH with higher predictive accuracy than taxonomic differences alone. Our findings underscore the roles of alpha-ketoglutarate, cysteine-glutathione disulfide, and N-acetylmethionine in NASH progression (Supplementary Figure 9). Upregulation of alpha-ketoglutarate is one of the most important metabolite predictors of NASH in our cohort and likely reflects the oxidative stress associated with the development of steatohepatitis from steatosis. Alpha-ketoglutarate is a derivative of glutamate that generates ATP in the TCA cycle, and serves as a substrate for intracellular glutathione (GSH) synthesis and turnover (Gaggini et al., 2018). In fatty liver disease, enhanced generation of reactive oxygen species leads to altered availability of GSH, a hepatic antioxidant that is perturbed in both humans and animal models of NASH (Nobili et al., 2005). The increased alpha-ketoglutarate in our children with NASH is likely an adaptive response to increased metabolic and oxidative stress in the liver. Moreover, we observed upregulation of gamma-glutamyltransferase (GGT), an enzyme that transaminates GSH, which likely reflects increased glutathione demand in the presence of hepatic inflammation and oxidative stress (Gaggini et al., 2018).

Current imaging and biomarker technologies have yet to demonstrate sufficient sensitivity and specificity to replace liver biopsy for distinguishing between simple steatosis and steatohepatitis as well as for staging fibrosis. Furthermore, though several studies have found elevated alpha-ketoglutarate in NASH, this biomarker alone has been unable to distinguish individuals with NAFL from the approximately one-third that will progress to NASH (Aragones et al., 2016). Using unbiased high-throughput metabolomics, we identified a panel of serum metabolite signatures of fatty liver disease and liver histology. Machine learning models identified key pathways predictive of NASH (predictive accuracy 97.1%), NAFL (80.4%) and histologically-confirmed pathologic injury (93.7%) in children and adolescents. We were able to distinguish NAFL from NASH with 90% accuracy using a combination of 15 metabolites including alpha-ketoglutarate, cysteine-glutathione disulfide, and N-acetylmethionine.

Interestingly, one 9 year-old obese Latina girl with dyslipidemia and pre-diabetes HgbA1C of 6.0 was clinically diagnosed with NAFL based on increased transaminases (ALT 143 U/L, AST 88 U/L, GGT 99 U/L) and steatosis on ultrasound. However, her plasma metabolite profile was most consistent with NASH, with substantially elevated alpha-ketoglutarate and decreased N-acetylmethionine (Figure 4). Indeed, 8 months after enrolling in the study, the subject underwent a liver biopsy that demonstrated histology consistent with NASH. It is uncertain whether the subject had undiagnosed NASH at enrollment, given the lack of biopsy at that time, though this is likely given her biochemical and clinical risk factors and the fact that only 18% of children have histologic progression to NASH over the course of 1–2 years (Xanthakos et al., 2017). This particular example demonstrates the powerful utility of our metabolite panel for the diagnosis of steatohepatitis, and the high predictive accuracy for monitoring fatty liver disease progression. Our study builds upon recent multi-omics studies of fatty liver disease in adult cohorts and highlights key differences in non-invasive biomarker profiles of NASH and fibrosis that may be specific to the pediatric population (Qin et al., 2010; Bajaj et al., 2015; Boursier et al., 2016; Loomba et al., 2017; Schwimmer et al., 2019).

A major limitation of this study is the small sample size of biopsy-proven NASH with histology. It is possible that some subjects classified as NAFL may have NASH, but have not yet undergone a diagnostic liver biopsy; as research-related liver biopsy pose ethical and medical risks, decision to biopsy remained with the patient’s clinician based on practice guidelines. Such cases would likely yield conservative performance estimates for our predictive models due to the unknown variation introduced by these misclassification events. The homogenous population may potentially limit generalizability. Some of the findings may reflect differences in BMI, although this was likely mitigated by inclusion of children with high BMI and normal aminotransaminases as the baseline for many of the analyses. Finally, our findings are strictly correlative; further studies defining the mechanisms by which gut microbiota and their metabolic activities mediate fatty liver disease are necessary to establish a causal relationship. Longitudinal sampling of at-risk youths across a spectrum of disease stages may help differentiate causal drivers of progression from underlying variance in genetic makeup or other predisposing factors. Despite these limitations, this study is to the best of our knowledge the largest cohort of Latino children and adolescents across the spectrum of fatty liver disease including biopsy-proven NASH. Our cohorts were well phenotyped, balanced in gender, and have been characterized by multiple -omics methods to yield a rich and detailed set of potential biomarkers and therapeutic targets. Large, multi-center, multi-ethnic studies are needed to validate the effect of microbiome-derived signatures and metabolites on fatty liver disease and progression of fibrosis.

In conclusion, our findings highlight perturbations of the oral and intestinal microbiota, imply microbial metagenomic alterations of metabolism, and identify distinctive metabolites associated with hepatic derangements in children with fatty liver disease. The ability of our classification models to predict disease classes as well as histologic findings supports the utility of metabolite profiling to function as a non-invasive method for prediction of NASH and fibrosis. Furthermore, the key biochemical and functional pathways identified in our models may represent clinically important therapeutic targets in a multi-prong approach to manage the spectrum of fatty liver disease starting in youth.

## Data Availability Statement

The datasets presented in this study can be found in online repositories. The names of the repository/repositories and accession number(s) can be found below: https://www.ncbi.nlm.nih.gov/, PRJNA480711 and http://doi.org/10.5281/zenodo.1467544.

## Ethics Statement

The studies involving human participants were reviewed and approved by CHLA Institutional Review Board, CHLA-15-00395. Written informed consent to participate in this study was provided by the participants’ legal guardian/next of kin.

## Author Contributions

KK, GA, NT, and FL analyzed the overall results data, wrote and edited the manuscript, and responsible for the final manuscript content. FL and DL performed statistical and bioinformatic analysis of the NGS data and edited the manuscript. JK reviewed and edited the final manuscript. KK and GA designed the research (project conception, development of research plan, and study oversight). KK, NC, MJ, and ML performed recruitment and collection of biological specimens and clinical data. SZ, MS, and CW performed NGS. All authors approved the final draft submission.

## Author Disclaimer

The content is solely the responsibility of the authors and does not necessarily represent the official views of the NIH.

## Conflict of Interest

KK performed this work while at CHLA and is currently affiliated with Novartis. JK was affiliated with Metabolon Inc., his expertise was sought to review the final manuscript after all analyses and interpretation were complete. The remaining authors declare that the research was conducted in the absence of any commercial or financial relationships that could be construed as a potential conflict of interest.

## Publisher’s Note

All claims expressed in this article are solely those of the authors and do not necessarily represent those of their affiliated organizations, or those of the publisher, the editors and the reviewers. Any product that may be evaluated in this article, or claim that may be made by its manufacturer, is not guaranteed or endorsed by the publisher.
