# Supplementary material for: Metabolomic Predictors of Non-alcoholic Steatohepatitis and Advanced Fibrosis in Children
Source: Front Microbiol. 2021 Aug 12;12:713234. doi: 10.3389/fmicb.2021.713234 (PMC8406633; doi:10.3389/fmicb.2021.713234)
Supplement: Supplementary file 1 [file Data_Sheet_1.PDF]

Figure S1. Random forests (RF) models of disease cohort using gut species level abundances. Related to Figure 1. (a) Receiver operating characteristic (ROC) curves of binary RF classification models for each group as indicated versus 'normal-BMI' baseline. (b) Distribution of BMI and species-level relative abundances for the features selected in the multiclass RF model (violin plots). RF model importance values are shown as shaded boxes with darker colors denoting higher mean importance.

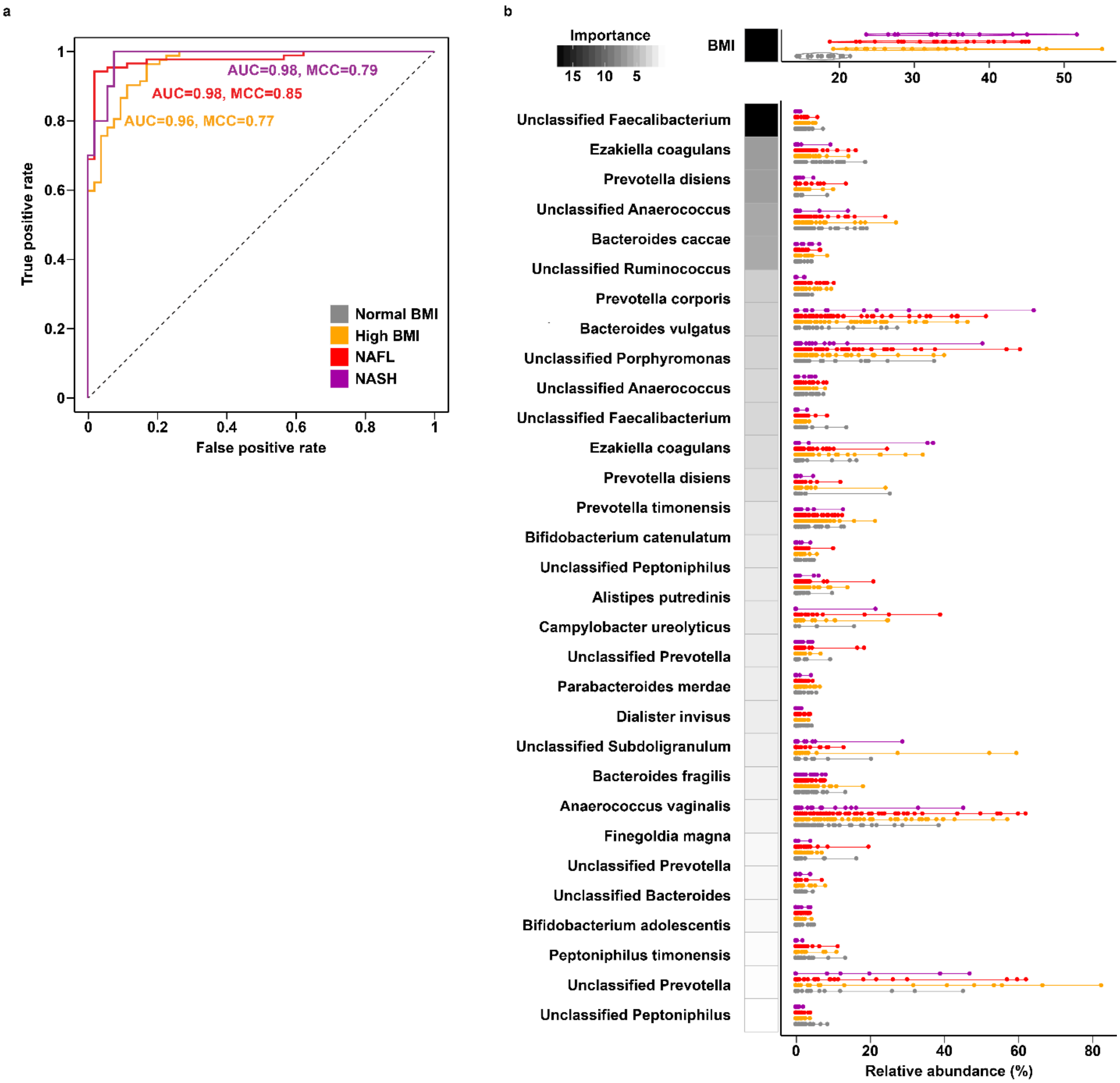

Figure S2. Oral microbiome composition in progressive fatty liver disease. Related to Figure 1. (a) Principal coordinates analysis (PCoA) plot of oral microbiota composition using Bray-Curtis distances. Ellipses show 95% confidence regions for each cohort. Percentages in brackets denote variation explained by each axis. (b) Boxplots show no significant differences in Shannon diversity (KW p=0.771) (top) and increased log-transformed *Bacteroidetes* to *Firmicutes* ratio (KW p=0.07498) (bottom) with advancing liver disease. (c) Receiver operating characteristic (ROC) curves of binary RF classification models for each group as indicated versus 'normal-BMI' baseline. (d) Distribution of BMI and genus-level relative abundances for the features selected in the multiclass RF model (violin plots). RF model importance values are shown as shaded boxes with darker colors denoting higher mean importance. (e) Heatmap of importance values for RF models of liver histology findings. Positive (red) and negative (blue) values indicate genera that are increased and decreased with higher grades of each finding, respectively. Asterisks (*) denote genera that are also significant (p<0.1) in ZINB regression analyses.

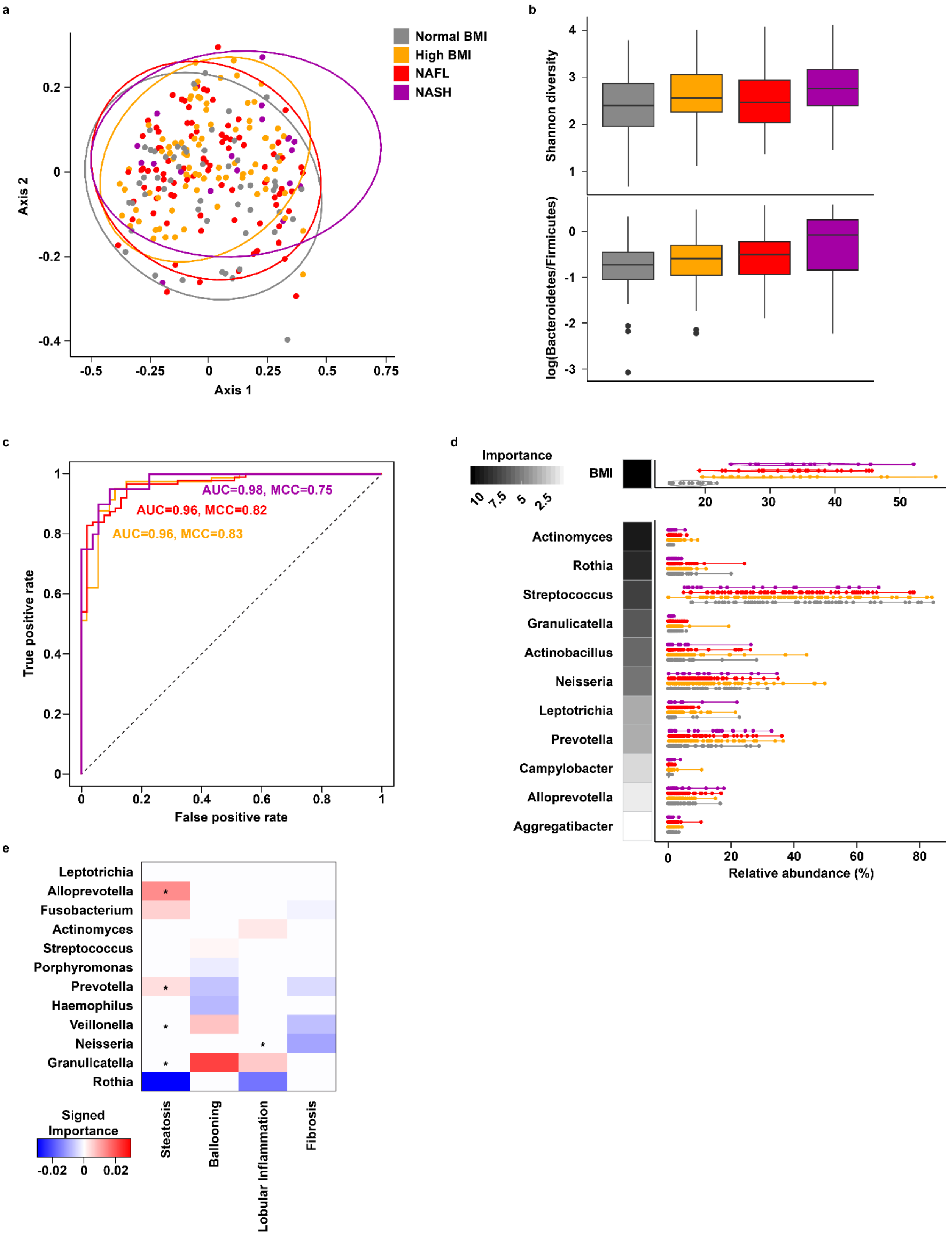
a
Normal BMI
High BMI
NAFL
NASH
Axis 2
Axis 1
b
Shannon diversity
log(Bacteroidetes/Firmicutes)
c
AUC=0.98, MCC=0.75
AUC=0.96, MCC=0.82
AUC=0.96, MCC=0.83
True positive rate
False positive rate
d
Importance
BMI
Actinomyces
Rothia
Streptococcus
Granulicatella
Actinobacillus
Neisseria
Leptotrichia
Prevotella
Campylobacter
Alloprevotella
Aggregatibacter
Relative abundance (%)
e
Leptotrichia
Alloprevotella
Fusobacterium
Actinomyces
Streptococcus
Porphyromonas
Prevotella
Haemophilus
Veillonella
Neisseria
Granulicatella
Rothia
Signed
Importance
Steatosis
Ballooning
Lobular Inflammation
Fibrosis

Figure S3. Gut microbiome composition in NASH (n=20) and normal-BMI (n=20) cohorts by shotgun metagenomics. Related to Figure 1.

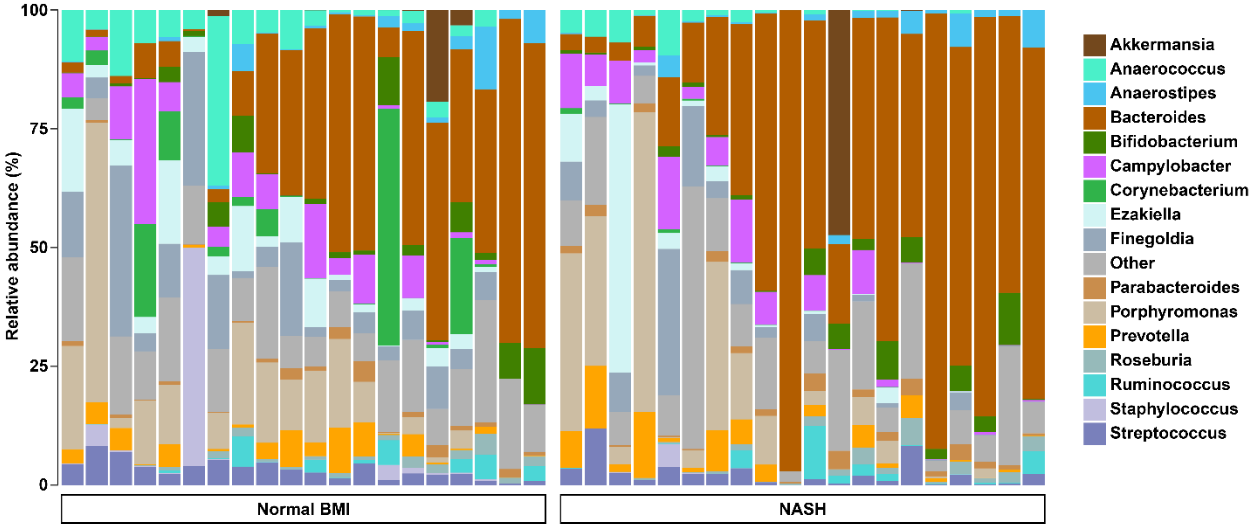

Figure S4. Taxonomic drivers of functional shifts in NASH. Related to Figure 1.

Overall enrichment of each KEGG pathway is denoted by an open diamond (◊). Taxa attenuating each functional shift are shown to the left of the vertical line, and taxa driving each functional shift are shown to the right of the vertical line. For each KEGG pathway, taxa shown along the top are increased in NASH and taxa shown along the bottom are decreased in NASH.

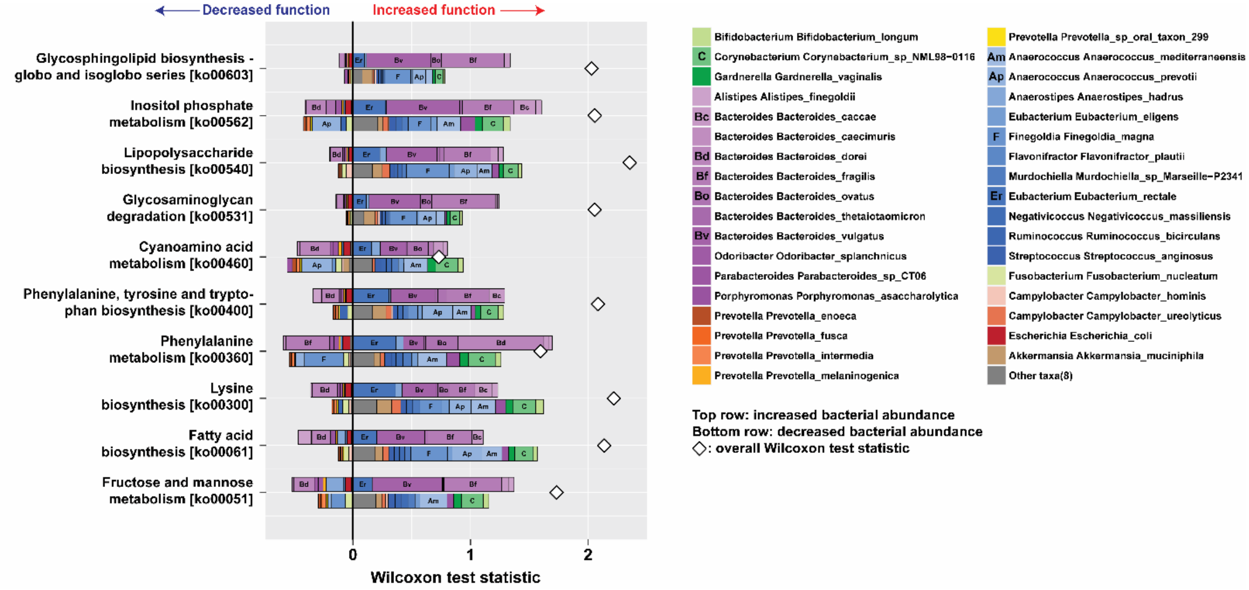

Figure S5. Plasma metabolite binary classification models against 'high-BMI' baseline. Related to Figure 3. Heatmap of importance values for RF models versus 'high-BMI' baseline. Shaded cells denote metabolites selected as features in each model. Red asterisks (*) denote metabolites identified as significantly altered in each group by linear regression.

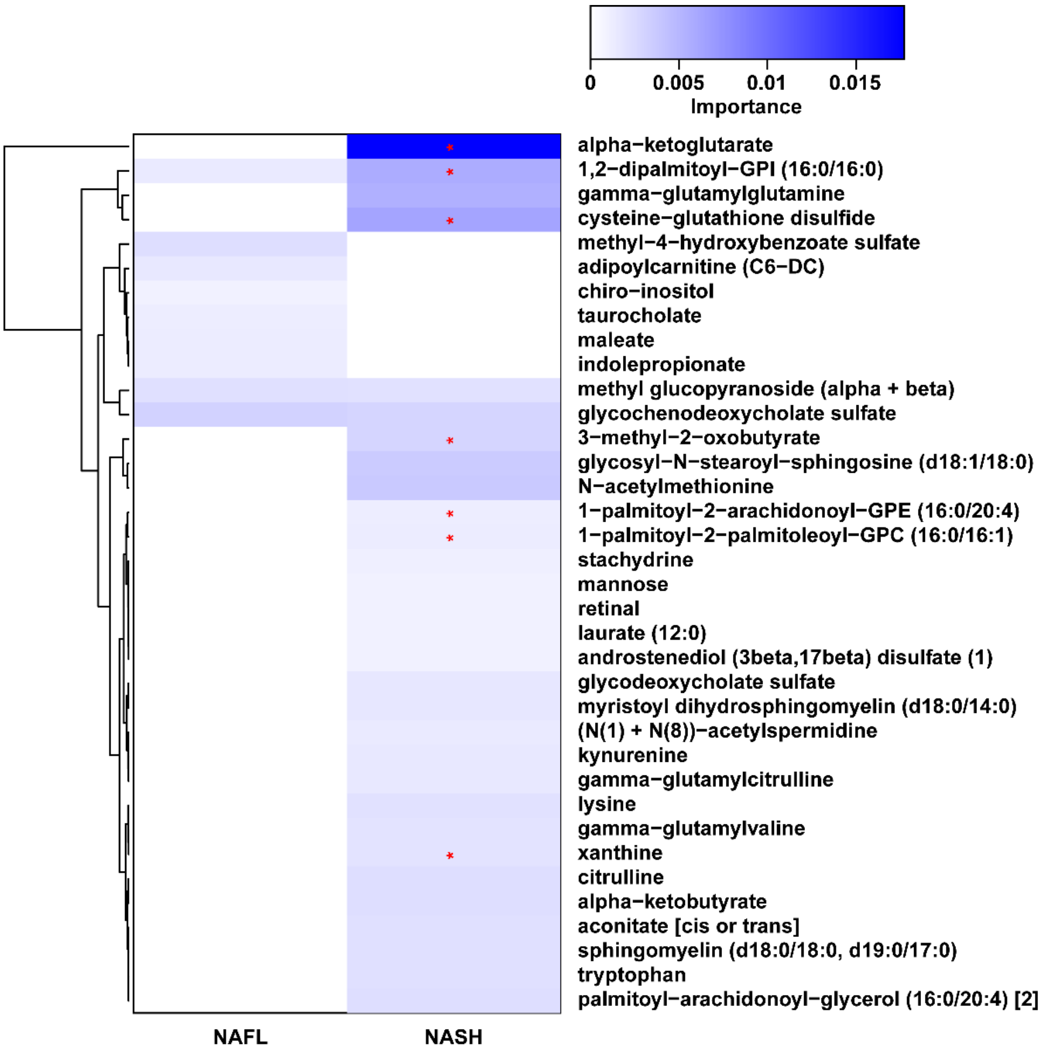

Figure S6. Fecal metabolite signatures of progressive fatty liver disease. Related to Figure 2. (a) t-SNE plot of fecal metabolite profiles. Ellipses show 95% confidence regions for each cohort. (b) Receiver operating characteristic (ROC) curves of binary RF classification models for each group as indicated versus ‘normal-BMI’ baseline. (c) Distribution of BMI and metabolite values for the features selected in the multiclass RF model (violin plots). RF model importance values are shown as shaded boxes with darker colors denoting higher mean importance. (d) Heatmap of importance values for RF models of liver histology findings. Positive (red) and negative (blue) values indicate metabolites that are increased and decreased with higher grades of each finding, respectively.

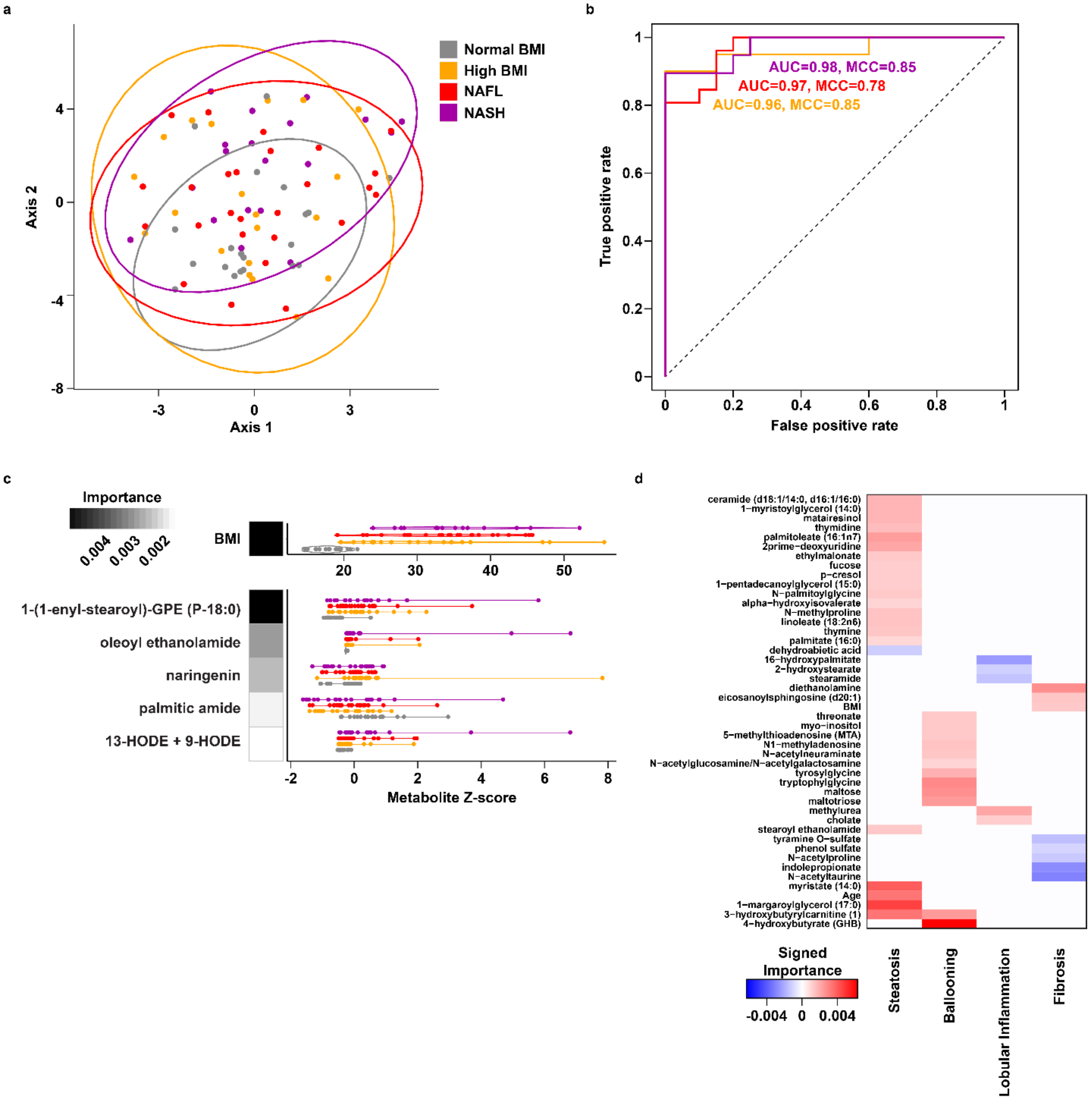

Figure S7. Fecal metabolite binary classification models against 'high-BMI' baseline. Related to Figure 3. (a) ROC curves of binary RF models for each group as indicated versus 'high-BMI' baseline. (b) Heatmap of importance values for RF models versus 'high-BMI' baseline. Shaded cells denote metabolites selected as features in each model.

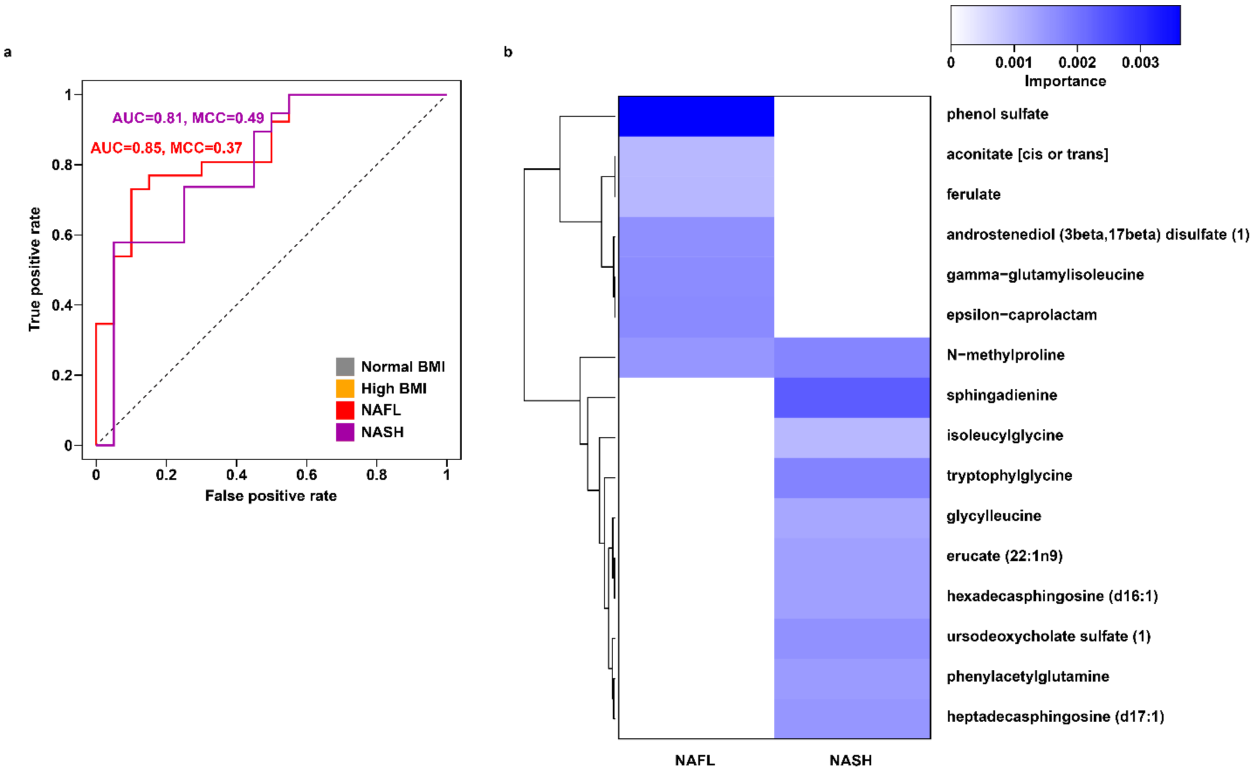

Figure S8. Plasma metabolite drivers of misclassification in histology findings. Related to Figure 4. Ground truth labels based on original clinical diagnosis for steatosis (a), lobular inflammation (b), and fibrosis (c) are shown along the x-axis. Predicted labels are shown as the indicated colors, and each point represents one individual.

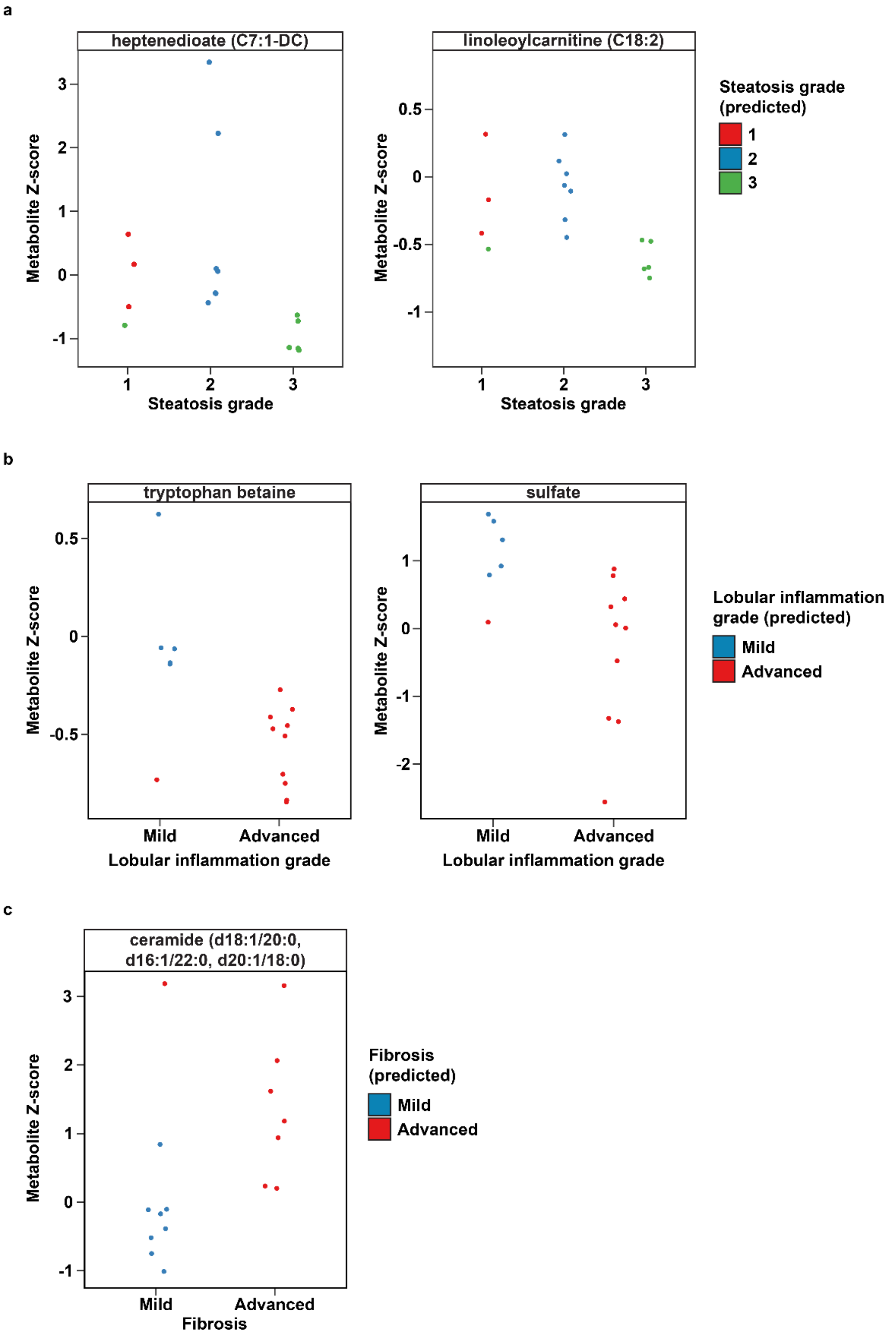

Figure S9. Relationships between alpha-ketoglutarate, cysteine-glutathione disulfide, and N-acetylmethionine. Related to all Figures. Alpha-ketoglutarate and cysteine-glutathione disulfide were increased (red text) in subjects with NASH, while N-acetyl-methionine was decreased (blue text). NAT = N-acetyl transferases, SAM = S-adenosylmethionine, SAH = *S*-Adenosyl-L-homocysteine, GLS = glutaminase, ALT = alanine transaminase, GS = glutathione synthetase, GSH = glutathione, GGT = gamma-glutamyltransferase, GSSG = glutathione disulfide, AA = amino acid, THF = tetrahydrofolate, cys = cysteine, gly = glycine

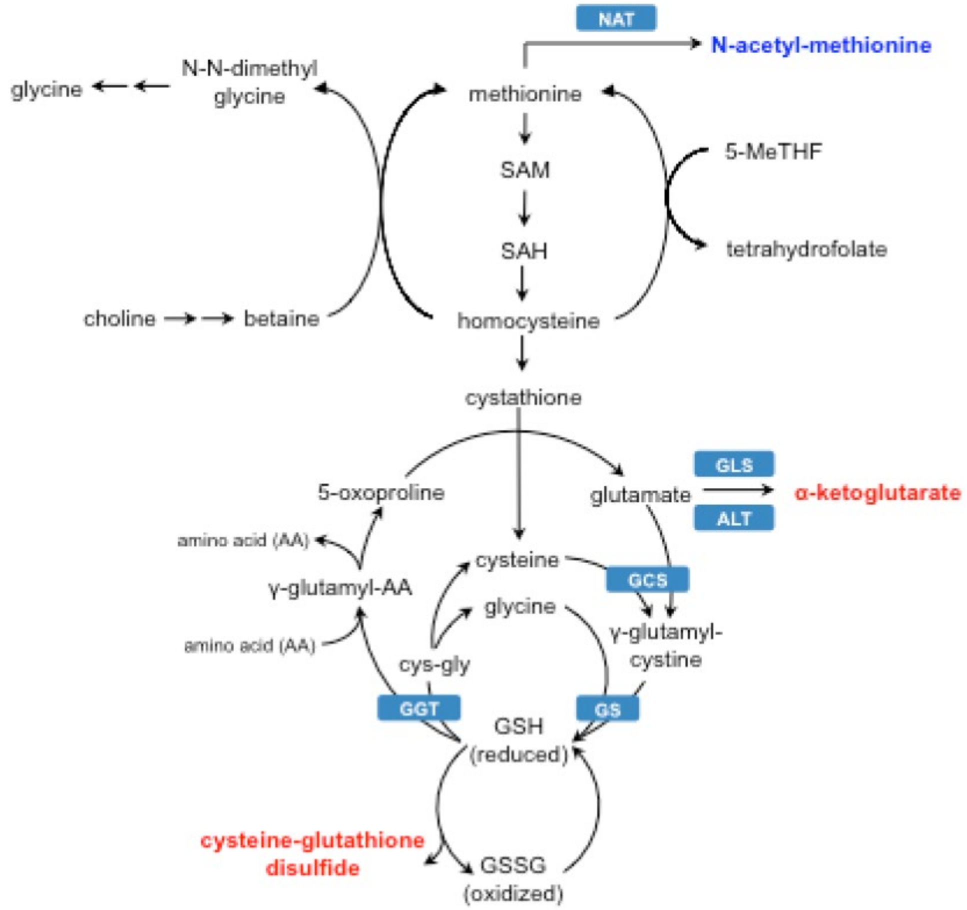

Table S1. PERMANOVA results for 16S microbiome profiling. Related to Figure 1.

**Rectal Swabs**		
**Bray-Curtis metric**		
**Variable**	**R^2**	**p-value**
Cohort	0.02251	0.024
Age	0.01030	0.017
Sex	0.02575	0.001
BMI	0.00450	0.542
Vitamin E	0.00400	0.748
Metformin	0.00462	0.568
**Jaccard metric**		
**Variable**	**R^2**	**p-value**
Cohort	0.02047	0.021
Age	0.00819	0.034
Sex	0.01760	0.001
BMI	0.00469	0.665
Vitamin E	0.00454	0.739
Metformin	0.00514	0.485
**JSD metric**		
**Variable**	**R^2**	**p-value**
Cohort	0.02516	0.025
Age	0.01278	0.014
Sex	0.03933	0.001
BMI	0.00479	0.476
Vitamin E	0.00330	0.763
Metformin	0.00400	0.648
**Oral Swabs**		
**Bray-Curtis metric**		
**Variable**	**R^2**	**p-value**
Cohort	0.02542	0.038
Age	0.01840	0.002
Sex	0.00702	0.189
BMI	0.00414	0.626
Vitamin E	0.00678	0.210
Metformin	0.00345	0.772
**Jaccard metric**		
**Variable**	**R^2**	**p-value**
Cohort	0.02262	0.026
Age	0.01524	0.003
Sex	0.00625	0.193
BMI	0.00486	0.524
Vitamin E	0.00691	0.147
Metformin	0.00434	0.673
**JSD metric**		
**Variable**	**R^2**	**p-value**
Cohort	0.02930	0.020

Age	0.02382	0.002
Sex	0.00972	0.072
BMI	0.00501	0.433
Vitamin E	0.00667	0.250
Metformin	0.00286	0.771

Table S2. Association between microbiome relative abundances and liver enzyme levels. Related to Figure 1.

Table S3. PERMANOVA results for shotgun microbiome profiling. Related to Figure 1.

Rectal Swabs		
**Bray-Curtis metric**		
**Variable**	**R^2**	**p-value**
Cohort	0.03508	0.159
Age	0.02703	0.319
Sex	0.05302	0.040
BMI	0.01157	0.933
**Jaccard metric**		
**Variable**	**R^2**	**p-value**
Cohort	0.03028	0.206
Age	0.02876	0.248
Sex	0.04363	0.048
BMI	0.01522	0.965
**JSD metric**		
**Variable**	**R^2**	**p-value**
Cohort	0.05023	0.088
Age	0.02040	0.523
Sex	0.06557	0.047
BMI	0.00706	0.887

Table S4. Differential taxa of shotgun data using DESeq2. Related to Figure 1.

Table S5. Differential abundance testing of microbially encoded pathways using DESeq2. Related to Figure 1.

Table S6. PERMANOVA results for metabolite profiling. Related to Figure 2.

**Plasma metabolites**		
**Variable**	**R^2**	**p-value**
BMI	0.05788	0.001
Age	0.02676	0.026
Sex	0.01903	0.323
Cohort	0.06994	0.018
Vitamin E	0.01614	0.542
Metformin	0.02555	0.067
**Fecal metabolites**		
**Variable**	**R^2**	**p-value**
BMI	0.04392	0.024
Age	0.01360	0.526
Sex	0.01669	0.306
Cohort	0.05361	0.282
Vitamin E	0.02597	0.160
Metformin	0.06164	0.018

Table S7. Random forests modeling of plasma and fecal metabolites. Related to Figure 2.

Table S8. Association between metabolite profiles and liver enzyme levels. Related to Figure 2.

Table S9. Confusion matrices for RF models of disease cohort and histology findings. Related to all Figures.

Cohort, fecal

		Predicted				
		**NMLBMI**	**ObeseNML**	**NAFLD**	**NASH**	**Class error (%)**
Truth	NMLBMI	18	1	1	0	10
	ObeseNML	2	7	8	3	65
	NAFLD	1	4	14	7	46.2
	NASH	0	5	10	4	78.9

Cohort, plasma

		Predicted				
		**NMLBMI**	**ObeseNML**	**NAFLD**	**NASH**	**Class error (%)**
Truth	NMLBMI	20	0	0	0	0
	ObeseNML	4	6	10	0	70
	NAFLD	1	8	14	3	46.2
	NASH	0	0	5	9	35.7

Diagnosis2, fecal

		Predicted		
		**BorderlineNASH**	**DefiniteNASH**	**Class error (%)**
Truth	BorderlineNASH	7	1	12.5
	DefiniteNASH	2	11	15.4

Steatosis2, fecal

		Predicted			
		**1**	**2**	**3**	**Class error (%)**
Truth	1	7	0	0	0
	2	2	7	0	22.2
	3	1	0	4	20.0

Fibrosis2, fecal

		Predicted		
		**Mild**	**Advanced**	**Class error (%)**
Truth	Mild	11	2	15.4
	Advanced	3	5	37.5

LobularInflammation2, fecal

		Predicted		
		**1**	**2**	**Class error (%)**
Truth	1	5	3	37.5
	2	1	12	7.7

Ballooning, fecal

		Predicted		
		**1**	**2**	**Class error (%)**
Truth	1	12	2	14.3
	2	2	5	28.6

Diagnosis2, plasma

		Predicted		
		**BorderlineNASH**	**DefiniteNASH**	**Class error (%)**
Truth	BorderlineNASH	3	2	40
	DefiniteNASH	0	11	0

Steatosis2, plasma

		Predicted			
		**1**	**2**	**3**	**Class error (%)**
Truth	1	3	0	1	25
	2	0	7	0	0
	3	0	0	5	0

Fibrosis2, plasma

		Predicted		
		**Mild**	**Advanced**	**Class error (%)**
Truth	Mild	8	1	11.1
	Advanced	0	7	0

LobularInflammation2, plasma

		Predicted		
		**1**	**2**	**Class error (%)**
Truth	1	5	1	16.7
	2	0	10	0

Ballooning, plasma

		Predicted		
		**1**	**2**	**Class error (%)**
Truth	1	8	1	11.1
	2	3	4	42.9
